# Using a New Circular Prediction Algorithm to Design an IMM Filter for Low Update Rate Radar System

**DOI:** 10.3390/s20185035

**Published:** 2020-09-04

**Authors:** Yung-Lung Lee

**Affiliations:** Department of Power Vehicle and Systems Engineering, Chung Cheng Institute of Technology, National Defense University, Taoyuan City 335, Taiwan; yunglunglee84@gmail.com

**Keywords:** circular predictor, maneuvering target tracking, nonlinear filters, Interacting Multiple Models (IMM) filter

## Abstract

For radar systems with low update rates; such as track-while-scan (TWS) systems using rotating phased array antennas; reducing the prediction error is a very important issue. A good interacting multiple models (IMM) hybrid filter combined with circular and linear filters that are defined in relation to three measurements has been proposed in the literature. However; the algorithm requires three previous measurements; and too much prior information will result in a reduced ability to predict the future position of a highly maneuvering target. A new circular prediction algorithm for maneuvering target tracking is proposed as a non-linear prediction filter in this paper. Based on this new predictor; we also proposed a new type of IMM filter that has good estimation performance for high maneuvering targets. The proposed hybrid filter is entirely defined in relation to two measurements in a three-dimensional space to obtain a better maneuver following capability than the three measurements hybrid filter. Two target profiles are included for a comparison of the performance of our proposed scheme with that of the conventional circular; linear and IMM filters. The simulation results show that under low update rates; the proposed filter has a faster and more stable estimation response than other filters

## 1. Introduction

Radar tracking capability is an important factor in the performance of weapon systems. Small size rotary phased array radars are widely used due to their light weight, low price, and high mobility. However, they must use a track-while-scan (TWS) method to track the target under low update rate conditions. The TWS radar system is a data-sampled filter whose tracking and prediction algorithms are based on previous observations that include measurement noise. A dynamic model and discrete-time data are needed for estimating and predicting the dynamic target. Hence, in order to achieve optimum target tracking performance, three key steps must be taken. First, detect the target immediately it commences the maneuvering process. Second, accurately estimate the lateral acceleration value of the target in as short time as possible. Third, the state estimate of the tracking filter must be correctly compensated according to the lateral acceleration value [1]. These steps illustrate that a good target tracking performance must be based on an exact mathematical model and a fast update rate. However, target tracking becomes quite difficult at low update rates. In general, a good mathematical model is key in determining target tracking performance, especially for low update rate radar systems. For this reason, implementing an appropriate mathematical model for optimal tracking performance is an important issue for low update radar systems.

The movement modes of the target are generally classified as maneuvering motion and non-maneuvering motion. The non-maneuvering motion refers to a linear motion at a uniform velocity. In addition, all other modes are maneuvering modes. The constant velocity (CV) model is usually used to describe non-maneuvering motion. The constant acceleration (CA) model is the simplest model for maneuvering targets. It can be used when the maneuver is small or random [2]. Some methods for solving this tracking problem have been proposed using a variety of approaches. One of these methods which utilizes a simple linear *α*–*β* filter for target tracking has been proposed in several studies [3,4,5]. In addition, a linear Kalman filter-based method is also used for trajectory estimation of a constant speed or constant acceleration target [6,7,8]. For the maneuvering target, these linear filters can achieve optimal filtering performance under fast update rate conditions. But at low update rate, the filtering performance of the linear filter will be significantly reduced.

A maneuvering target acceleration model with exponential autocorrelation acceleration was also developed [9]. The model treats the acceleration as a disturbance on a constant velocity trajectory. If the straight line hypothesis is relaxed, the model is the result of the coordinated turn [10,11]. Thus, the coordinated turn can be further simplified to a kinematics problem [12,13]. However, the main problem of the coordinated turn model is that it is a set of non-linear coupled equations and, therefore, difficult to solve.

Another geometry-based circular prediction method, known as center point approach (CAP), has been introduced to address tracking problems. The CAP is based on a polar coordinate system whose origin is the center of the circle. It must define a circle from previous measurements, and then obtain a circular trajectory by calculating the center of the circle and radius. Because the calculation of center coordinates is very complicated and polar coordinates may appear discontinuous when switching, CAP is not suitable for further analysis of prediction stability, uncertainty, and performance. In order to solve these problems, a three-point circular prediction (3PC) algorithm that does not require calculation of center and radius in relative coordinates has been proposed [14,15]. The prediction states are completely defined by the three points used to construct the circle. The 3PC algorithm simplifies the prediction process and can be assisted by the dynamic scheduler to improve tracking performance. Furthermore, the 3PC algorithm constrains the target motion to a circular trajectory on a smooth curve. However, the actual situation of target motion can be approximated by circular and straight piecewise curves. In addition, since the 3PC algorithm requires three previous measurements, too much prior information will result in a poorer ability to predict the future position of the highly maneuvering target. Therefore, the estimation performance will be reduced when only the circular filter is used. For these reasons, some circular filters are integrated with traditional filters that include a linear target model [16]. An interacting multiple model (IMM) algorithm is an effective method for the integration of multiple filters. This algorithm can be used to estimate the target state by combining multiple models with various filters. In the IMM algorithm, the target model is selected based on the Markov transition probability matrix and the estimate is obtained by weighting the filtered values of different target models. Therefore, the combination of the target model and the filter determines the performance of the IMM estimator [17,18,19,20]. The most basic IMM estimator uses two target models, one for constant velocity moving targets with little change and the other for maneuvering targets with varying acceleration.

Further research was carried out and it was found that target tracking using a multi-radar system [21,22,23,24,25,26,27] or compound sensor system (integrated radar and optical sensor system) could compensate for the poor tracking performance of radars with low update rates. However, the above system has the disadvantages that a single radar cannot be used alone and the detection distance of the optical sensor is short. Therefore, there is still an urgent need to improve the target tracking capability of a single radar system under the condition of a low update rate.

This paper focuses on the development of a three-dimensional tracking algorithm that constrains the predicted state to a circular and linear path for a low update rate radar system. The proposed method can predict the circular and tangent motion of a maneuvering target with fewer previous measurements than 3PC. The method can also design an integrated hybrid filter based on these two prediction rules. It is expected that the maneuvering target can be effectively estimated at low update rates.

The remainder of this paper is organized as follows. The planar circular and tangent-tracking algorithms in a local coordinate system are introduced in Section 2. The three-dimensional tracking algorithm is proposed in Section 3. A new hybrid filter design by combining circular and linear predictor is illustrated in Section 4. The performances of different filters are verified by two benchmark trajectories, and the simulation results are presented in Section 5. Finally, the conclusions and future works are described in Section 6.

## 2. The Planar Circular and Tangent Motion Model

**Figure 1:**
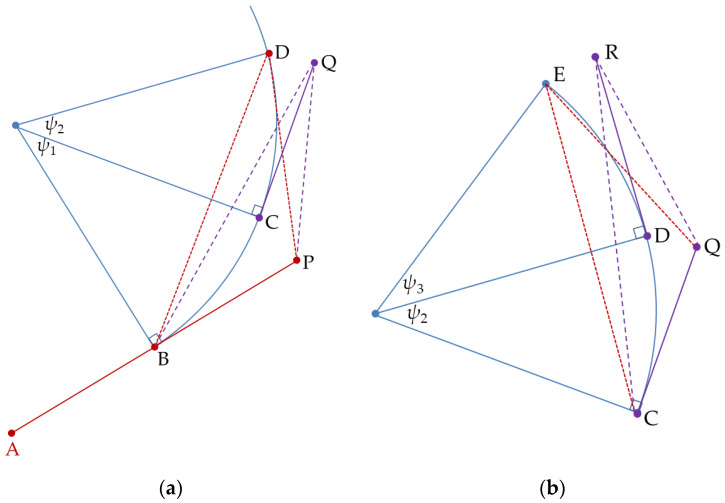
Estimated geometric relationship, (**a**) initial relationship; (**b**) subsequent relationship.

In order to solve the disadvantages of the three-point-measurements circular (3PC) algorithm which may lead to a slow estimation response performance, this paper proposed a two-point-measurements circular and tangent (2PCT) prediction algorithm. The estimated geometric relationship is shown in Figure 1. The algorithm proposed in this paper will distinguish two parts; initial calculation and subsequent calculation. For initial calculation, points A and B are the two positions of the target detected by radar at the beginning. Because only two measurements are known, it is not possible to determine the target’s motion pattern. Assuming that the target moves linearly from position A to B, then, position P can be estimated using linear prediction (α-β filter). When the radar detects the third position C of the target, and if point C is far from the position P, it can be detected that the target is in a circular motion. The target’s future position D of the circular motion and the future position Q of the tangent motion can be estimated based on points B and C on the target circular trajectory and position P on the linear trajectory, as shown in Figure 1a. For subsequent calculations, the future position for circular motion (point E) or tangential motion (Point R) can be estimated using the same initial calculation, as long as the two positions (Points C and D) on the trajectory are measured, as shown in Figure 1b. These algorithms are derived as follows:

### 2.1. Two-Point Circular Prediction (2PC)

**Figure 2:**
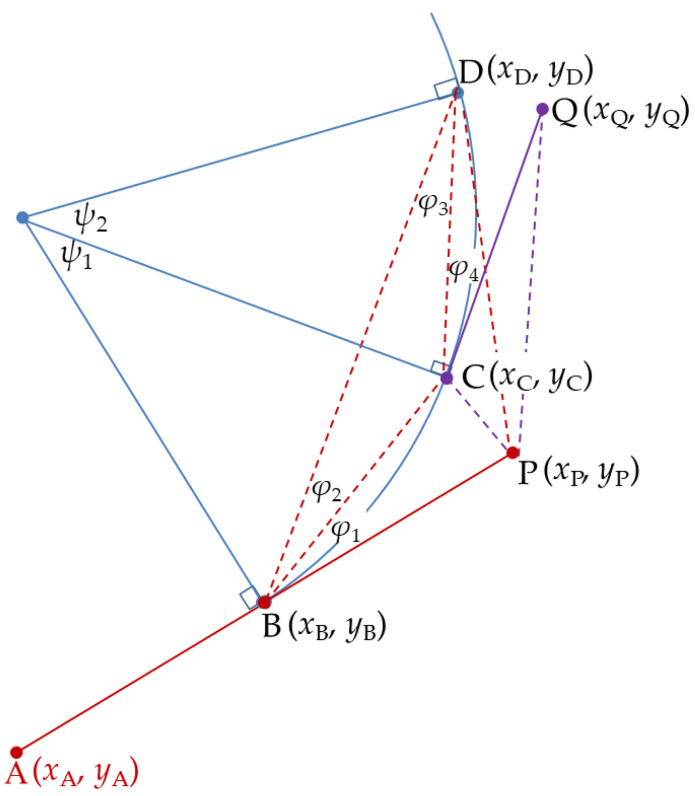
Properties of three points lying on a circle.

By considering the position of points C and P relative to point B, a Cartesian coordinate system labeled *x* and *y* with its origin at point B can be introduced as shown in Figure 2. Equations (1)–(4) define the distances in terms of *x*_i_ and *y*_i_.

(1) $$R_{\mathrm{BD}}^{2}=x_{D}^{2}+y_{D}^{2}$$

(2) $$R_{\mathrm{BP}}^{2}=x_{P}^{2}+y_{P}^{2}=x_{A}^{2}+y_{A}^{2}$$

(3) $$R_{\mathrm{CD}}^{2}={\left(x_{D}-x_{C}\right)}^{2}+{\left(y_{D}-y_{C}\right)}^{2}$$

(4) $$R_{\mathrm{PD}}^{2}={\left(x_{D}-x_{P}\right)}^{2}+{\left(y_{D}-y_{P}\right)}^{2}$$

**Figure 3:**
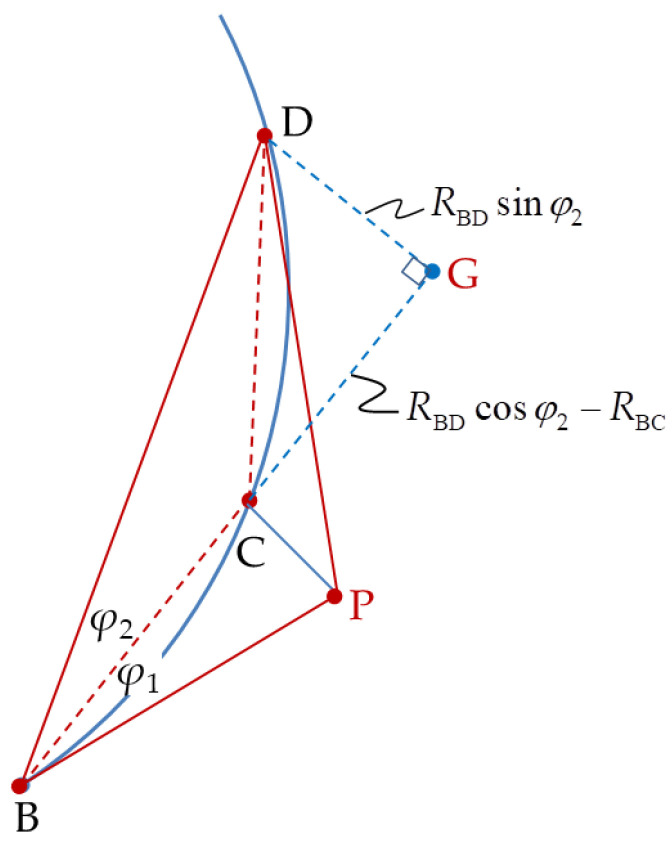
Triangle ∆BCD extension to triangle ∆BGD.

**Figure 4:**
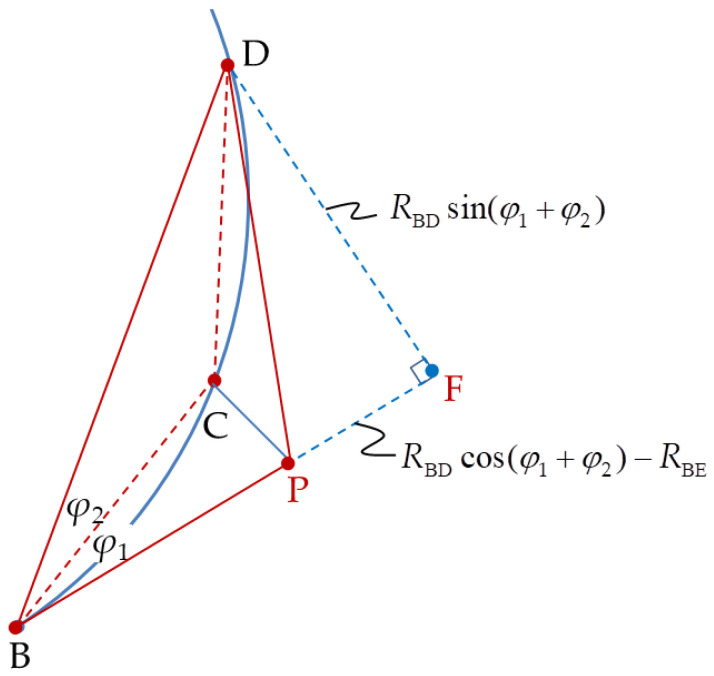
Triangle ∆BPD extension to triangle ∆BFD.

Equations (3) and (4) can be rewritten as:

(5) $$x_{D}x_{C}+y_{D}y_{C}=\frac{R_{\mathrm{BD}}^{2}+R_{\mathrm{BC}}^{2}-R_{\mathrm{CD}}^{2}}{2}$$

(6) $$x_{D}x_{P}+y_{D}y_{P}=\frac{R_{\mathrm{BD}}^{2}+R_{\mathrm{BP}}^{2}-R_{\mathrm{PD}}^{2}}{2}$$

where *R*_CD_ and *R*_PD_ can be obtained from ∆CGD in Figure 3 and ∆PFD in Figure 4, respectively.

(7) $$R_{\mathrm{CD}}^{2}=R_{\mathrm{BD}}^{2}+R_{\mathrm{BC}}^{2}-2R_{\mathrm{BD}}R_{\mathrm{BC}}\cos\varphi_{2}$$

(8) $$R_{\mathrm{PD}}^{2}=R_{\mathrm{BD}}^{2}+R_{\mathrm{BP}}^{2}-2R_{\mathrm{BD}}R_{\mathrm{BP}}\cos(\varphi_{1}+\varphi_{2})$$

Taking into account Equations (7) and (8) and solving Equations (5) and (6) for the relative position [*x*_D_ *y*_D_]^T^, we obtain the desired relationship.

(9) $$\left[\begin{array}{c} x_{D}\\ y_{D} \end{array}\right]={\left[\begin{array}{cc} x_{C} & y_{C}\\ x_{P} & y_{P} \end{array}\right]}^{-1}\left[\begin{array}{c} R_{\mathrm{BC}}\cos\varphi_{2}\\ R_{\mathrm{BP}}\cos(\varphi_{1}+\varphi_{2}) \end{array}\right]R_{\mathrm{BD}}$$

**Figure 5:**
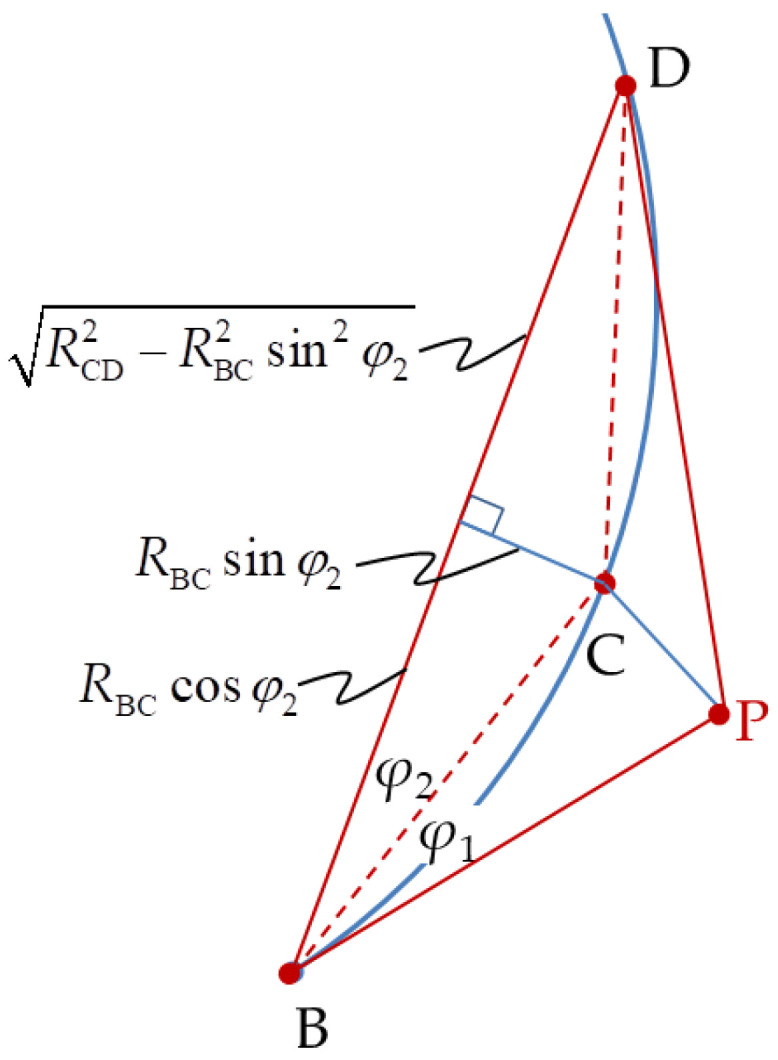
Triangle ∆BCD side length relationship.

The unknown distance, *R*_BD_, in Equation (9) is determined from ∆BCD in Figure 5.

(10) $$R_{\mathrm{BD}}=R_{\mathrm{BC}}\left[\cos\varphi_{2}+\sqrt{{\left(\frac{R_{\mathrm{CD}}}{R_{\mathrm{BC}}}\right)}^{2}-\sin^{2}\varphi_{2}}\right]$$

From Figure 2 and by using the cosine rule, Equation (10) can be rewritten as:

(11) $$R_{\mathrm{BD}}=R_{\mathrm{BC}}\left[\cos\varphi_{2}+\sin\varphi_{2}\sqrt{\frac{1}{\sin^{2}\varphi_{3}}-1}\right]$$

Note that Equation (11) is also valid for straight line maneuvers. If target is at a constant speed (CS) angular turn, that is $\varphi_{1}=\varphi_{2}=\varphi_{3}$, Equation (7) reduces to:

(12) $$R_{\mathrm{BD}}^{\mathrm{CS}}=R_{\mathrm{BC}}\left[\cos\varphi_{1}+\sqrt{1-\sin^{2}\varphi_{1}}\right]$$

If the target is under a straight line maneuvering condition:

(13) $$\underset{\varphi_{1}\to 0}{\lim}R_{\mathrm{BD}}^{\mathrm{CS}}=2R_{\mathrm{BC}}=2R_{\mathrm{BP}}$$

In order to extend the proposed algorithm from two to three dimensions, the prediction, Equation (9), can be interpreted as a vector quantity. Define the vector ${\vec{r}}_{\mathrm{BC}}$ of length *R*_BC_ pointing from point B towards point C, and vector ${\vec{r}}_{\mathrm{BP}}$, respectively by considering the cross product performed in a two-dimensional plane.

(14) $$\left\|{\vec{r}}_{\mathrm{BC}}\times {\vec{r}}_{\mathrm{BP}}\right\|=R_{\mathrm{BC}}R_{\mathrm{BP}}\sin\varphi_{1}$$

Equation (9) can be rewritten as:

(15) $$\left[\begin{array}{c} x_{D}\\ y_{D} \end{array}\right]=T(\pi /2){\vec{n}}_{\mathrm{BP}}\frac{\cos\varphi_{2}}{\sin\varphi_{1}}R_{\mathrm{BD}}+T(-\pi /2){\vec{n}}_{\mathrm{BC}}\frac{\cos(\varphi_{1}+\varphi_{2})}{\sin\varphi_{1}}R_{\mathrm{BD}}$$

where the matrix ***T***(*α*) is the rotational transformation matrix defined as:

(16) $$T(\alpha )=\left[\begin{array}{cc} \cos\alpha & \sin\alpha \\ -\sin\alpha & \cos\alpha \end{array}\right]$$

${\vec{n}}_{\mathrm{BP}}$ is a unit vector in the ${\vec{r}}_{\mathrm{BP}}$ direction. The new position prediction Equation (15) is composed of the rotation and scaling unit vectors ${\vec{n}}_{\mathrm{BP}}$ and ${\vec{n}}_{\mathrm{BC}}$ in the two-dimensional plane. The same rotation must be performed according to the normal vector defined in the two-dimensional plane when the new prediction position equation is derived in the three-dimensional plane.

### 2.2. Two-Point Tangent Prediction (2PT)

Analogous to two-points-circular prediction in Section 2.1, and observing Figure 2, the following can be obtained:

(17) $$R_{\mathrm{BQ}}^{2}=x_{Q}^{2}+y_{Q}^{2}$$

(18) $$R_{\mathrm{BP}}^{2}=x_{P}^{2}+y_{P}^{2}$$

(19) $$R_{\mathrm{CQ}}^{2}={\left(x_{Q}-x_{C}\right)}^{2}+{\left(y_{Q}-y_{C}\right)}^{2}$$

(20) $$R_{\mathrm{PQ}}^{2}={\left(x_{Q}-x_{P}\right)}^{2}+{\left(y_{Q}-y_{P}\right)}^{2}$$

**Figure 6:**
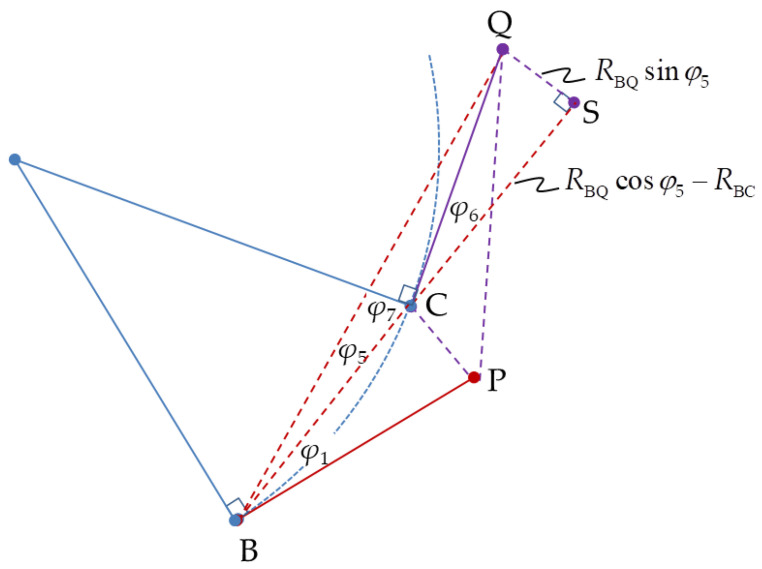
Triangle ∆BCQ extension to triangle ∆BSQ.

**Figure 7:**
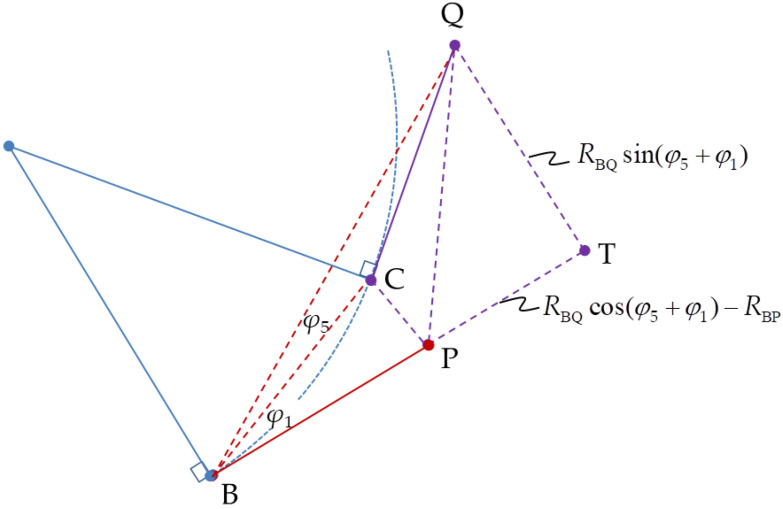
Triangle ∆BPQ extension to triangle ∆BTQ.

Equations (19) and (20) can be rewritten as:

(21) $$x_{Q}x_{C}+y_{Q}y_{C}=\frac{R_{\mathrm{BQ}}^{2}+R_{\mathrm{BC}}^{2}-R_{\mathrm{CQ}}^{2}}{2}$$

(22) $$x_{Q}x_{P}+y_{Q}y_{P}=\frac{R_{\mathrm{BQ}}^{2}+R_{\mathrm{BP}}^{2}-R_{\mathrm{PQ}}^{2}}{2}$$

where *R*_CQ_ and *R*_PQ_ can be obtained from ∆CSQ in Figure 6 and ∆PTQ in Figure 7, respectively.

(23) $$R_{\mathrm{CQ}}^{2}=R_{\mathrm{BQ}}^{2}+R_{\mathrm{BC}}^{2}-2R_{\mathrm{BQ}}R_{\mathrm{BC}}\cos\varphi_{5}$$

(24) $$R_{\mathrm{PQ}}^{2}=R_{\mathrm{BQ}}^{2}+R_{\mathrm{BP}}^{2}-2R_{\mathrm{BQ}}R_{\mathrm{BP}}\cos(\varphi_{1}+\varphi_{5})$$

Taking into account Equations (23) and (24) and solving Equations (21) and (22) for the relative position [*x*_Q_ *y*_Q_]^T^, we obtain the desired relationship.

(25) $$\left[\begin{array}{c} x_{Q}\\ y_{Q} \end{array}\right]={\left[\begin{array}{cc} x_{C} & y_{C}\\ x_{P} & y_{P} \end{array}\right]}^{-1}\left[\begin{array}{c} R_{\mathrm{BC}}\cos\varphi_{5}\\ R_{\mathrm{BP}}\cos(\varphi_{1}+\varphi_{5}) \end{array}\right]R_{\mathrm{BQ}}$$

The unknown distance, *R*_BD_, in Equation (9) is determined from ∆BCQ in Figure 6.

(26) $$\begin{array}{ll} R_{\mathrm{BQ}}^{2} & =R_{\mathrm{CQ}}^{2}+R_{\mathrm{BC}}^{2}-2R_{\mathrm{BC}}R_{\mathrm{CQ}}\cos(\pi /2+\varphi_{7})\\ & =R_{\mathrm{CQ}}^{2}+R_{\mathrm{BC}}^{2}+2R_{\mathrm{BC}}R_{\mathrm{CQ}}\sin(\varphi_{7}) \end{array}$$

Equations (25) can be rewritten as:

(27) $$R_{\mathrm{BQ}}=R_{\mathrm{BC}}\sqrt{1+\frac{R_{\mathrm{CQ}}^{2}}{R_{\mathrm{BC}}^{2}}+2\frac{R_{\mathrm{CQ}}}{R_{\mathrm{BC}}}\sin(\varphi_{7})}$$

When the target is under a constant speed (CS) angular turn condition, that is $\varphi_{6}=\varphi_{1}$, $\varphi_{7}=\pi /2-\varphi_{1}$, and $R_{\mathrm{BP}}=R_{\mathrm{CQ}}$, Equation (27) can be presented as:

(28) $$R_{\mathrm{BQ}}^{\mathrm{CS}}=R_{\mathrm{BC}}\sqrt{1+\frac{R_{\mathrm{BP}}^{2}}{R_{\mathrm{BC}}^{2}}+2\frac{R_{\mathrm{BP}}}{R_{\mathrm{BC}}}\cos(\varphi_{1})}$$

From Figure 6, we obtain that $R_{\mathrm{CQ}}\sin(\varphi_{6})=R_{\mathrm{BQ}}\sin(\varphi_{5})$, that is,

(29) $$\varphi_{5}=\sin^{-1}\left(\frac{R_{\mathrm{CQ}}\sin(\varphi_{6})}{R_{\mathrm{BQ}}}\right)=\sin^{-1}\left(\frac{R_{\mathrm{CQ}}\sin(\varphi_{1})}{R_{\mathrm{BC}}\sqrt{2+2\cos(\varphi_{1})}}\right)$$

and in case of a straight-line maneuver, $\varphi_{1}=0$, we obtain:

(30) $$\underset{\varphi_{1}\to 0}{\lim}R_{\mathrm{BQ}}^{\mathrm{CS}}=2R_{\mathrm{BP}}$$

Analogous to Equation (15) in Section 2.1, the prediction Equation (25) can be rewritten in the vector form as:

(31) $$\left[\begin{array}{c} x_{Q}\\ y_{Q} \end{array}\right]=T(\pi /2){\vec{n}}_{\mathrm{BP}}\frac{\cos\varphi_{5}}{\sin\varphi_{1}}R_{\mathrm{BQ}}+T(-\pi /2){\vec{n}}_{\mathrm{BC}}\frac{\cos(\varphi_{1}+\varphi_{5})}{\sin\varphi_{1}}R_{\mathrm{BQ}}$$

## 3. Three-Dimensional Motion Estimation

The targets in the real world are to operate in three-dimensional spaces and, consequently, the trackers cannot be constrained to make predictions in two dimensions. Because the two-dimensional plane is formed by three points, the plane is, in fact, still in a three-dimensional space. Therefore, the estimation algorithms can be performed on this two-dimensional plane and then converted back to the global three-dimensional coordinate system.

The 2PC and 2PT prediction algorithms described in the previous section were calculated based on two measurements and one predictive value. These three positions define two vectors, ${\vec{r}}_{\mathrm{BC}}$ and ${\vec{r}}_{\mathrm{BP}}$, as shown in Figure 2. These two vectors are important parameters in the transformation between the two-dimensional plane and three-dimensional space. The normal vector ${\vec{n}}_{S}$ of a two-dimensional plane can be obtained by cross-product calculation for the normalized ${\vec{r}}_{\mathrm{BC}}$ and ${\vec{r}}_{\mathrm{BP}}$. When three vectors form a right-handed coordinate system, the normal vector direction is positive, which can also be determined by the right-handed screw rule that rotates ${\vec{r}}_{\mathrm{BC}}$ to the direction of ${\vec{r}}_{\mathrm{BP}}$. The transformation of the circular predictive plane from the original Cartesian coordinate system can be achieved by a multi-step rotation of the coordinate system. The first step is to rotate about the *z*–axis so that the projection of the normal vector onto the *x*–*y* plane is aligned with the *x*–axis. A three-dimensional transformation matrix, ***G***_1_(*φ*_s_), can be used to perform this rotation, where *φ*_s_ is the polar rotation angle of the normal vector projected onto the *x*–*y* plane.

(32) $$G_{1}(\varphi_{s})=\left[\begin{array}{ccc} \cos\varphi_{s} & \sin\varphi_{s} & 0\\ -\sin\varphi_{s} & \cos\varphi_{s} & 0\\ 0 & 0 & 1 \end{array}\right]$$

The second step is to rotate the coordinate system about the transformed *y*–axis after aligning with the *x*–axis. This rotation can be performed by rotation matrix ***G***_2_(*ϑ*_s_), where *ϑ*_s_ is the direction cosine of the normal vector relative to the *z*–axis.

(33) $$G_{2}(\vartheta_{s})=\left[\begin{array}{ccc} \cos\vartheta_{s} & 0 & -\sin\vartheta_{s}\\ 0 & 1 & 0\\ \sin\vartheta_{s} & 0 & \cos\vartheta_{s} \end{array}\right]$$

The combined coordinate transformation can be obtained by constructing the rotation matrix:

(34) $$G=G_{1}(\varphi_{s})G_{2}(\vartheta_{s})$$

Considering a zero *z*–component including the predicted position and the normalized vector, Equation (16) can be rewritten as:

(35) $$T(\alpha )=\left[\begin{array}{ccc} \cos\alpha & \sin\alpha & 0\\ -\sin\alpha & \cos\alpha & 0\\ 0 & 0 & 1 \end{array}\right]$$

Substituting the vector described in the relative Cartesian coordinates for the normalized vector, Equation (15) can be written as:

(36) $$\left[\begin{array}{c} x_{D}\\ y_{D}\\ 0 \end{array}\right]=T(\pi /2)G\left[\begin{array}{c} {x^{\prime }}_{P}\\ {y^{\prime }}_{P}\\ {z^{\prime }}_{P} \end{array}\right]\frac{R_{\mathrm{BD}}}{R_{\mathrm{BP}}}\frac{\cos\varphi_{2}}{\sin\varphi_{1}}+T(-\pi /2)G\left[\begin{array}{c} {x^{\prime }}_{C}\\ {y^{\prime }}_{C}\\ {z^{\prime }}_{C} \end{array}\right]\frac{R_{\mathrm{BD}}}{R_{\mathrm{BC}}}\frac{\cos(\varphi_{1}+\varphi_{2})}{\sin\varphi_{1}}$$

where ***G*** can be obtained from Equation (34). The circular prediction of Equation (36) is described with respect to a Cartesian coordinates system where the *x*–*y* plane coincides with the circular prediction plane. Including the back transformation into Equation (36):

(37) $$\left[\begin{array}{c} {x^{\prime }}_{D}\\ {y^{\prime }}_{D}\\ {z^{\prime }}_{D} \end{array}\right]=G^{-1}\left[\begin{array}{c} x_{D}\\ y_{D}\\ 0 \end{array}\right]$$

involves the evaluation of the matrix product:

(38) $$ℜ=G^{-1}T(\pi /2)G$$

Note that since $T(-\pi /2)=T^{T}(\pi /2)G$ and ***G*** is orthogonal, then the combined transformation matrix, $G^{-1}T(-\pi /2)G={ℜ}^{T}$. Therefore, Equation (36) can be written with respect to the global Cartesian coordinate system as:

(39) $$\left[\begin{array}{c} {x^{\prime }}_{D}\\ {y^{\prime }}_{D}\\ {z^{\prime }}_{D} \end{array}\right]=ℜ\left[\begin{array}{c} {x^{\prime }}_{P}\\ {y^{\prime }}_{P}\\ {z^{\prime }}_{P} \end{array}\right]s_{1}+{ℜ}^{T}\left[\begin{array}{c} {x^{\prime }}_{C}\\ {y^{\prime }}_{C}\\ {z^{\prime }}_{C} \end{array}\right]s_{2}$$

where,

(40) $$s_{1}=\frac{R_{\mathrm{BD}}}{R_{\mathrm{BP}}}\frac{\cos\varphi_{2}}{\sin\varphi_{1}}$$

(41) $$s_{2}=\frac{R_{\mathrm{BD}}}{R_{\mathrm{BC}}}\frac{\cos(\varphi_{1}+\varphi_{2})}{\sin\varphi_{1}}$$

Adding the coordinates of the first point and replacing the relative coordinates with global coordinates, we obtain the circular prediction in global Cartesian coordinates:

(42) $$\left[\begin{array}{c} x_{D}\\ y_{D}\\ z_{D} \end{array}\right]=ℜ\left[\begin{array}{c} x_{P}\\ y_{P}\\ z_{P} \end{array}\right]s_{1}+{ℜ}^{T}\left[\begin{array}{c} x_{C}\\ y_{C}\\ z_{C} \end{array}\right]s_{2}+(I-ℜs_{1}-{ℜ}^{T}s_{2})\left[\begin{array}{c} x_{B}\\ y_{B}\\ z_{B} \end{array}\right]$$

Equation (42) is a two-point circular (2PC) three-dimensional prediction. Similarly, the two-point tangential (2PT) three-dimensional prediction is as follows:

(43) $$\left[\begin{array}{c} x_{Q}\\ y_{Q}\\ z_{Q} \end{array}\right]=ℜ\left[\begin{array}{c} x_{P}\\ y_{P}\\ z_{P} \end{array}\right]s_{3}+{ℜ}^{T}\left[\begin{array}{c} x_{C}\\ y_{C}\\ z_{C} \end{array}\right]s_{4}+(I-ℜs_{3}-{ℜ}^{T}s_{4})\left[\begin{array}{c} x_{B}\\ y_{B}\\ z_{B} \end{array}\right]$$

where,

(44) $$s_{3}=\frac{R_{\mathrm{BQ}}}{R_{\mathrm{BP}}}\frac{\cos\varphi_{5}}{\sin\varphi_{1}}$$

(45) $$s_{4}=\frac{R_{\mathrm{BQ}}}{R_{\mathrm{BC}}}\frac{\cos(\varphi_{1}+\varphi_{5})}{\sin\varphi_{1}}$$

According to the aforementioned 2PCT algorithms, the prediction steps in this work are described as follows:

Step 1: Assuming linear motion from positions A to B, the linear prediction position P can be obtained by linear prediction, as shown in Figure 1a.

Step 2: If the position at the next time-step of position B, obtained through the measurement, does not fall on the linear prediction position P but on position C, the target is judged to be in the maneuver flight state of the circular motion. The 2PCT prediction rules mentioned in this article can be used at this point.

Step 3: Calculate position D by the 2PC prediction method according to Equation (42), and calculate position Q by the 2PT prediction method according to Equation (43).

Step 4: Using the 2PT prediction position Q as a reference, if the next time-step position measurement of position C occurs at position Q, it means that the target moves in a straight line after position C; if the next time-step position measurement of position C appears at position D, it means the target is in a circular motion state after position C.

Step 5: Based on the predicted position Q calculated by the 2PT algorithm and the position measurements C and D, the predicted position E at the next time-step is calculated by the 2PC algorithm, according to Equation (42). Then, the next time-step predicted position R is calculated by the 2PT algorithm, according to Equation (43), as shown in Figure 1b.

Step 6: Repeat Step 3 to Step 5 until the tracking mission is finished.

As can be seen from the aforementioned prediction steps, the 2PCT prediction algorithm proposed in this work can grasp the target near the linear prediction position and the circular prediction position in each prediction stage. It can reduce the probability of the target escaping from the tracking. In addition, in each prediction step, only two measurement values and one prediction value are required. Compared with the 3PC algorithm, fewer measurement values were used to predict the future position of the target. Therefore, it can be more effective in tracking high-maneuvering targets.

For illustration purposes, the measurement error has not been considered in the calculation procedure of the algorithm proposed in this chapter; therefore all the calculation steps are described by the measurement value and the predicted value. If the measurement error is considered, the “filtered value” and the “predicted value” will be used for calculation. Therefore, in consideration of the influence of measurement error, the third point is regarded as the maneuvering flight state of the target if the filtered value obtained after measurement and calculation does not fall at the predicted point P. In addition, under real conditions, the system must have measurement errors; that is, there will be a certain degree of difference between the filtered value and the predicted value at point P. Therefore, the algorithm proposed in this manuscript will enter step 3 after the third point and consider the calculation process of the circular prediction and tangent prediction at the same time.

## 4. Interacting Multiple Models (IMM) Estimation Algorithm

The tracking filter plays a key role in accurate estimation and prediction of maneuvering target’s states. The prediction algorithms developed in 3PC and 2PC circular prediction are appropriate for circle like trajectories. However, the actual trajectory can be approximated by a segmented curve of a circle and a straight line, so the performance of the standalone circular filter may be reduced. Therefore, integrating the circular filters and traditional linear filters is needed. An IMM hybrid filter provides an effective solution to this problem.

The IMM filter algorithm integrates different target motion models and considers process noise and measurement noise to solve the problem of maneuvering target tracking. Among the standard IMM filters, the M1 filter and the M2 filter are usually selected as the second-order and third-order Kalman filters respectively. In other words, M1 is used when the target is moving at or nearly at a fixed speed, while M2 is suitable for maneuvering targets. The total filter value is calculated by weighting the filter value of different models. The transition probability between different models is controlled by the Markov Process. The operation of the IMM filter algorithm is explained as follows [28].

Step 1: Model interaction

After interaction, the initial state estimation ${\hat{X}}_{0j}(k-1/k-1)$ and error covariance $P_{0j}(k-1/k-1)$ of the *j* model are calculated according to the sub-filters of different models.

(46) $${\hat{X}}_{0j}\left(k-1/k-1\right)=\sum_{i=1}^{n}{\hat{X}}_{i}\left(k-1/k-1\right)\mu_{i/j}\left(k-1/k-1\right)$$

(47) $$\begin{array}{l} P_{0j}\left(k-1/k-1\right)=\sum_{i=1}^{n}\mu_{i/j}\left(k-1/k-1\right)\times \\ \left\{P_{i}(k-1/k-1)+\left[{\hat{X}}_{i}(k-1/k-1)-{\hat{X}}_{0j}(k-1/k-1)\right]{\left[{\hat{X}}_{i}(k-1/k-1)-{\hat{X}}_{0j}(k-1/k-1)\right]}^{T}\right\} \end{array}$$

where *i*, *j* = 1,...,*n*, *n* is the model quantity, and the mixing probabilities are:

(48) $$\mu_{j}(k-1/k-1)=\frac{\varphi_{ij}\mu_{i}(k-1)}{\sum_{i=1}^{n}\varphi_{ij}\mu_{i}(k-1)}$$

where $\mu_{i}(k-1)$ is the model probability of the *i* model at *k*–1 time and $\varphi_{ij}$ is the model transition probability. ${\hat{X}}_{i}(k-1/k-1)$ and $P_{i}(k-1/k-1)$ are the state estimation and error covariance of *i* model, respectively.

Step 2: Filtering algorithm

${\hat{X}}_{0j}(k-1/k-1)$ and $P_{0j}(k-1/k-1)$ are used to compute the state estimate, ${\hat{X}}_{i}(k/k)$, and error covariance, $P_{i}(k/k)$, of the *j* model.

(49) $${\hat{X}}_{j}\left(k/k-1\right)=A_{j}{\hat{X}}_{0j}\left(k-1/k-1\right)$$

(50) $$P_{j}\left(k/k-1\right)=A_{j}P_{0j}\left(k-1/k-1\right)A_{j}^{T}+Q_{j}$$

(51) $$s_{j}\left(k\right)=H_{j}P_{j}\left(k/k-1\right)H_{j}^{T}+R_{j}$$

(52) $$K_{j}=P_{j}\left(k/k-1\right)H_{j}^{T}s_{j}^{-1}\left(k\right)$$

(53) $$P_{j}\left(k/k\right)=\left(I-K_{j}H_{j}\right)P_{j}\left(k/k-1\right)$$

(54) $${\bar{Z}}_{j}\left(k\right)=Z\left(k\right)-H_{j}{\hat{X}}_{j}\left(k/k-1\right)$$

(55) $${\hat{X}}_{j}\left(k/k\right)={\hat{X}}_{j}\left(k/k-1\right)+K_{j}{\bar{Z}}_{j}\left(k\right)$$

where ***X***(*k*) is the system state; ***Z***(*k*) is the system measurement; ***P***(*k*) is the error covariance; ***K*** is the Kalman Gain; ***s***(*k*) is innovation covariance; $\bar{Z}(k)$ is the residual innovation sequence; ***Q*** is the program noise covariance; and ***R*** is the measurement noise covariance.

Step 3: Compute likelihood function

It is computed by the following equation:

(56) $$\Lambda_{j}\left(k\right)={\left|2\pi s_{j}(k)\right|}^{-1/2}\times \exp\left\{\frac{-1}{2}{\left[{\bar{Z}}_{j}(k)\right]}^{T}{\left[s_{j}(k)\right]}^{-1}\left[{\bar{Z}}_{j}(k)\right]\right\}$$

The parameter *π* in Equation (56) is the ratio of the circumference of the circle to its diameter.

Step 4: Model probability update

(57) $$\mu_{j}(k)=\frac{\Lambda_{j}(k)\sum_{i=1}^{n}\varphi_{ij}\mu_{i}(k-1)}{\sum_{j=1}^{r}\Lambda_{j}(k)\sum_{i=1}^{n}\varphi_{ij}\mu_{i}(k-1)}$$

Step 5: Combination

The total state estimate is obtained by a weighted sum of the estimates from the sub-filters of different models.

(58) $$\hat{X}(k/k)=\sum_{j=1}^{n}{\hat{X}}_{j}(k/k)\mu_{j}(k)$$

(59) $$P\left(k/k\right)=\sum_{j=1}^{n}\mu_{j}\left(k/k\right)\times \{P_{j}(k/k)+\left[{\hat{X}}_{j}(k/k)-\hat{X}(k/k)\right]{\left[{\hat{X}}_{j}(k/k)-\hat{X}(k/k)\right]}^{T}\}$$

In this paper, the Kalman filter is used for the linear prediction models. In addition, 3PC, 2PC, and 2PT prediction models with closed-form solutions are implemented in the form of an extended Kalman filter. Two types of hybrid IMM filters are introduced, namely, the 3PCL IMM filter, which consists of the 3PC and linear filter, and the 2PCT IMM filter, which is integrated by the 2PC and 2PT.

**Figure 8:**
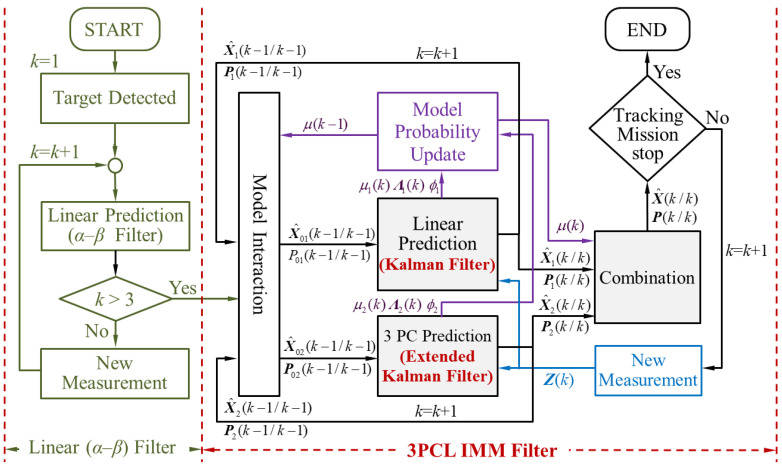
The 3PCL IMM filter algorithm flow chart.

The algorithm flow-chart of the 3PCL IMM filter is shown in Figure 8. The entire operation process can be divided into two parts, namely: simple linear filtering and IMM filtering. When the number of target position samples is less than three points, the linear filtering method can only be used, because the number of target information required for the circular prediction calculation has not been satisfied. In addition, the proposed algorithm uses linear prediction (*α*–*β* filter) to track the target when k≤3. It is assumed that there is only one target in the calculation process, and the trajectory correlation is not considered. When the target position samples are more than three points, the IMM hybrid filtering operation can be started.

**Figure 9:**
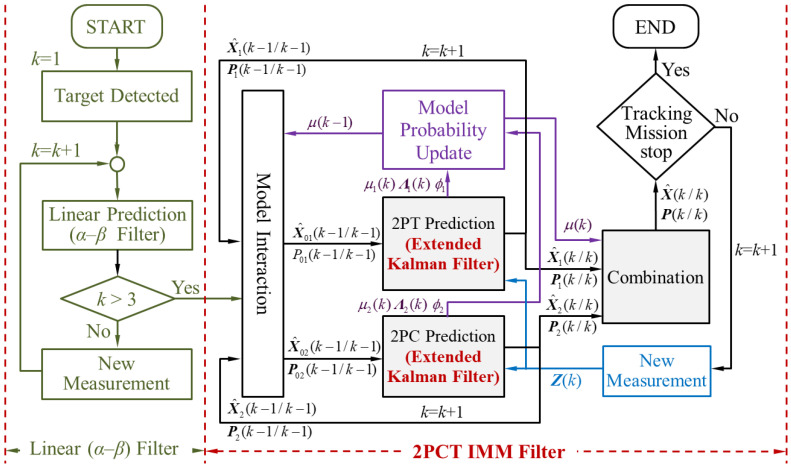
The 2PCT IMM filter algorithm flow chart.

The 2PCT IMM filter has an interactive multiple model algorithms that combine the linear and circular prediction algorithms. The traditional IMM filter integrates linear prediction and circular prediction, in which the linear prediction algorithm predicts the target position at the next moment based on the linear equation formed by target positions at the previous and current moment. The circular prediction algorithm predicts the target position at the next moment based on the three-point circular prediction algorithm, which is composed of the information of the target position at the first two moments and the current moment. The 2PCT algorithm proposed in this manuscript is a combination of the linear and circular prediction algorithms. In the linear prediction, based on the changing trend of the target positions at the previous and current moment, the target position at the next moment is predicted in the tangent direction. In the circular prediction, based on the changing trend of the target positions at the previous and current moments, the target position at the next moment is predicted in the circular motion direction. The linear and circular prediction algorithms can predict the target position at the next moment in a comprehensive manner by relying less on past target information and instead considering the target movement trend, so as to avoid the influence of past position information on the prediction ability of the maneuvering target position. In this manuscript, the IMM algorithm is used to integrate the 2PT tangent prediction and the 2PC circular prediction of the proposed 2PCT algorithm, so as to give it a good prediction ability for maneuvering targets, which is also the main contribution of this manuscript. The algorithm process is shown in Figure 9.

## 5. Simulation and Discussion

In this section, different filters are assessed for a maneuvering target tracking problem. In order to verify the estimated performance of the filter for different types of maneuvering targets, this paper designs two types of maneuvering target scenarios, namely the slow scouting flight (Scenario 1) and the fast attacking flight (Scenario 2). The flight conditions for each scenario are described as follows:

(1) Scenario 1: Scenario 1 is composed of two trajectories in different directions of circular motion. The target is at the initial position (*x*, *y*, *z*) = (6000 m, 4500 m, 1000 m) to make a scouting flight with a lateral acceleration of 3.5 G and −7 G at a speed of Mach 0.85. The time at which the lateral acceleration changes is 20 s and the total flight time is 40 s.
(2) Scenario 2: The target is at the initial position (*x*, *y*, *z*) = (20,000 m, 20,000 m, 100 m), flying at Mach 2.0, and making a turn with 30 G of lateral acceleration at four way points (16,000 m, 16,000 m, 100 m), (9000 m, 18,000 m, 100 m), (12,000 m, 2000 m, 100 m), and (4000 m, 4000 m, 100 m).

In practice, the update rate of the radar ranges from 4.0 to 0.2 Hz. A general mechanical scanning radar has a low update rate (usually less than 1.0 Hz). An electronically scanned array radar has a faster update rate that can go even higher than 10 Hz. Therefore, an update rate higher than 1.0 Hz is called a fast update rate, and an update rate lower than 1.0 Hz is called a low update rate. In this paper, low update rate radar is taken as the research subject. In order to highlight how the proposed algorithm can effectively deal with the problems faced by a low update rate radar in tracking maneuvering targets, the update rate was set at 0.5 Hz (sampling time is *t*_s_ = 2) during the simulation verification.

The measurement noise covariance is *σ*^2^ = 100. The tracking performance is evaluated by computing the maximum estimation error (ME) and root mean square error (RMSE) for the true target position, compared to the predicted state position.

### 5.1. Estimation Performance Test for Scenario 1

**Figure 10:**
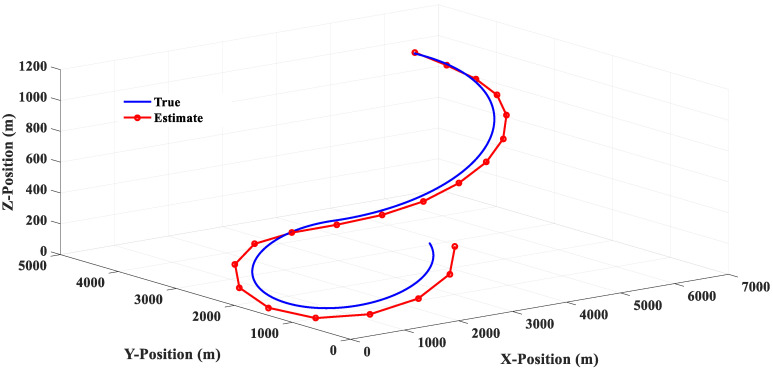
Linear (*α*–*β* filter) estimation response (Scenario 1).

**Figure 11:**
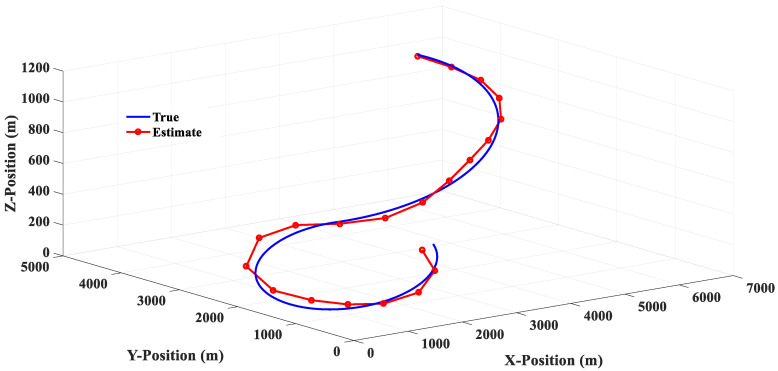
The 3PC filter estimation response (Scenario 1).

**Figure 12:**
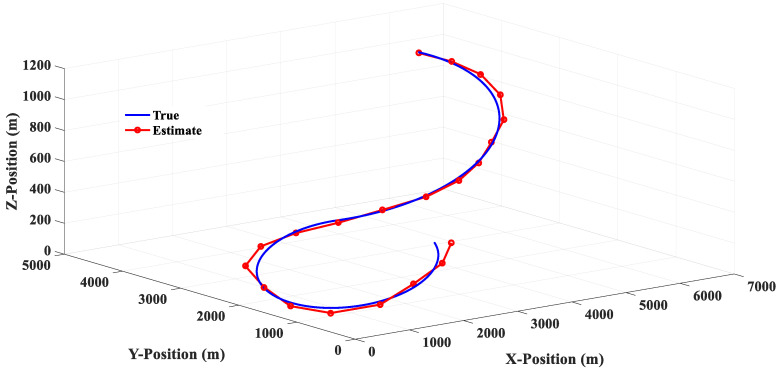
The 3PCL IMM filter estimation response (Scenario 1).

**Figure 13:**
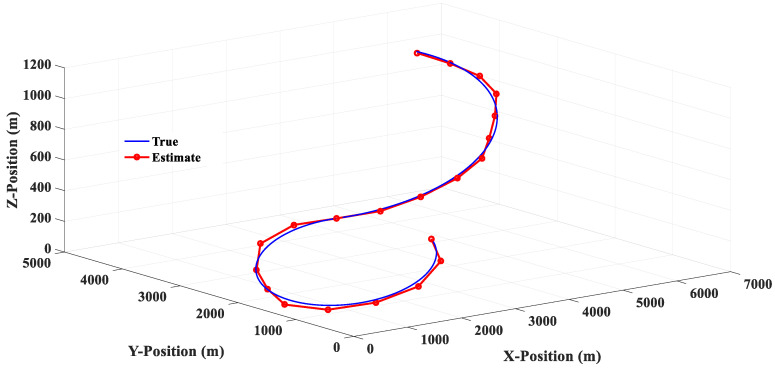
The 2PCT IMM filter estimation response (Scenario 1).

**Figure 14:**
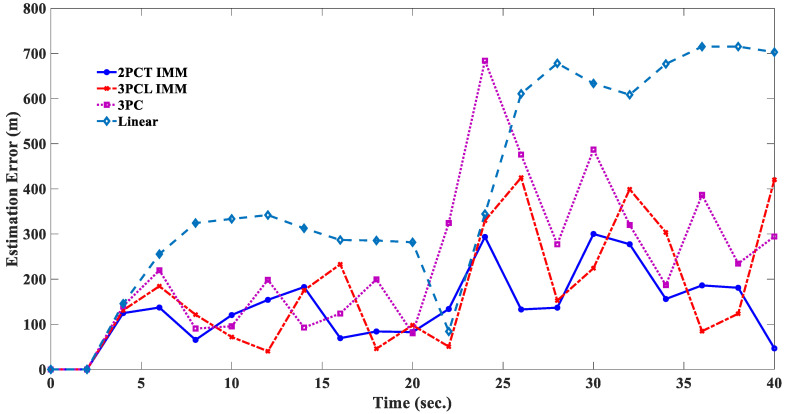
Comparison of estimated errors of different filters (Scenario 1).

The estimated responses of different filters to scenario 1 are shown in Figure 10, Figure 11, Figure 12 and Figure 13. Figure 14 shows the estimated errors of different filters. The *α*–*β* filter is a first-order (linear) filter. In the calculation, the position of the next moment is predicted linearly according to the position of the previous moment and the current moment. Although the calculation mechanism is relatively simple, a steady-state tracking error is generated when tracking a nonlinear moving target, as shown by the simulation results in Figure 10. Figure 14 also shows that when the lateral acceleration of the target is 3.5 G and −7.0 G respectively, the steady-state tracking error of the *α*-*β* filter is about 350 m and 700 m respectively, which indicates that the greater the lateral acceleration is, the greater the steady-state tracking error of the *α*–*β* filter will be.

The simulation results in Figure 11 show that the 3PC filter uses the position information of the first two moments and the current moment to make a circular prediction of the target position at the next moment; there will be no steady-state tracking error for the target with a circular motion, but the estimated response will show obvious oscillations near the real trajectory. It is also found that when the lateral acceleration of the target is 3.5 G and −7.0 G, the greater the target lateral acceleration is, the greater the estimated response oscillation will be. In addition, Figure 14 shows that when the target suddenly changes the turning direction at the 20th second, the 3PC filter will cause a large tracking error in the estimation of the target position at the 22nd and 24th second, which means that the algorithm of the 3PC filter relies too heavily on previous information and loses the ability to predict the rapid changes of the future state, which is a significant disadvantage of the 3PC filter.

The 3PCL IMM filter is an Interacting Multiple Models (IMM) filter combined with a linear and 3PC filter, and the tracking response is shown in Figure 12. By comparing Figure 10, Figure 11 and Figure 12, it can be seen that the 3PCL IMM filter can not only avoid the linear filter’s defect of being prone to producing a steady-state tracking error but also reduce the degree of oscillation of the 3PC filter’s estimation response. In addition, it can be seen from Figure 14 that the 3PCL IMM filter can also prevent tracking errors from occurring when the target suddenly changes the turning direction. By comparing the estimation error changes of the 3PC and 3PCL IMM filters in Figure 14, we can see that the estimation response of the 3PCL IMM filter is less oscillatory than that of the 3PC filter, but the oscillation is still obvious. This is because the 3PCL IMM filter still contains the algorithm mechanism of the 3PC filter, therefore the 3PCL IMM filter relies too heavily on previous information and loses its ability to predict the rapid change of future states.

The 2PCT IMM filter has interactive multiple model algorithms that combine the linear and circular prediction algorithms. The linear predictive algorithm is a tangent predictive algorithm, while the circular predictive algorithm is a two-point circular predictive algorithm. The comparison between Figure 12 and Figure 13 shows that the estimation response of the 2PCT IMM filter is less oscillatory than that of the 3PCL IMM filter. Figure 14 also shows that the estimation error of the 2PCT IMM filter varies slightly. This shows that the proposed 2PCT IMM filter has better target-tracking performance compared with other traditional filters.

### 5.2. Estimation Performance Test for Scenario 2

**Figure 15:**
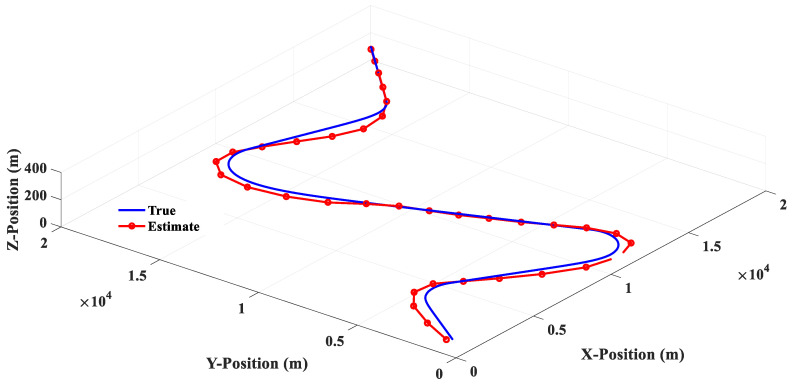
Linear (*α*–*β* filter) estimation response (Scenario 2).

**Figure 16:**
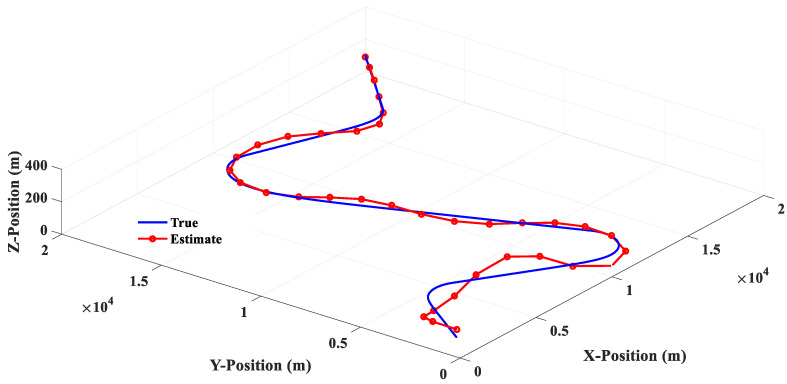
The 3PC filter estimation response (Scenario 2).

**Figure 17:**
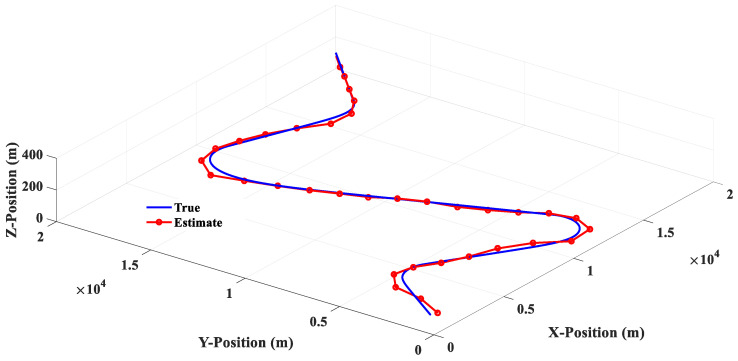
The 3PCL IMM filter estimation response (Scenario 2).

**Figure 18:**
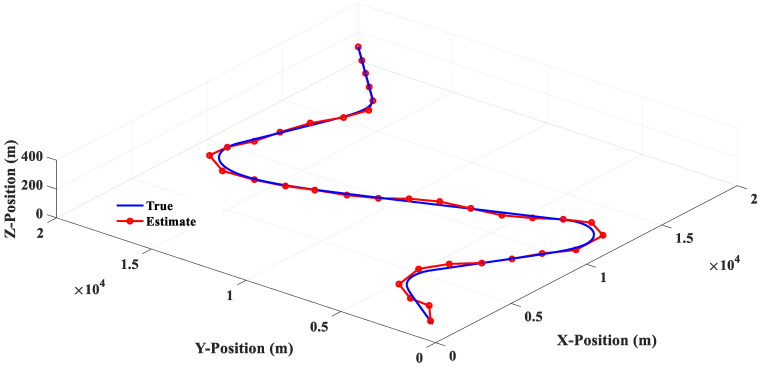
The 2PCT IMM filter estimation response (Scenario 2).

**Figure 19:**
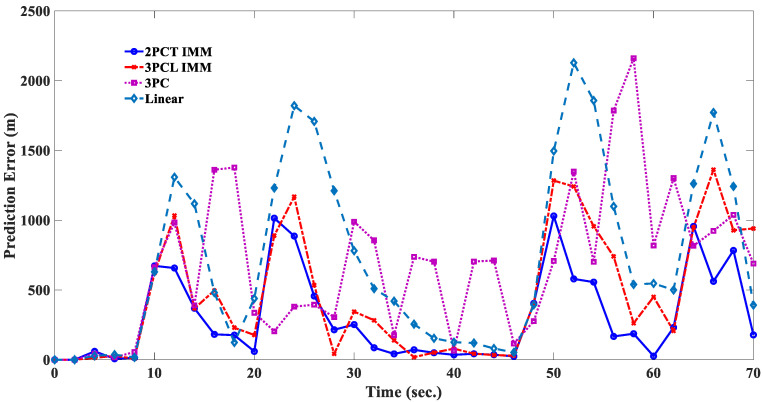
Comparison of estimated errors of different filters (Scenario 2).

Scenario 2 is composed of four groups of straight lines combined with turning trajectories. Figure 15 shows that the linear filter has a good estimation performance for linear motion, but there is a large estimation error when the target makes a turn. Figure 16 shows that the estimation response of 3PC circular filter will oscillate when the target trajectory is changed from linear motion to circular motion, and then to linear motion. This phenomenon is due to the fact that 3PC circular filter needs to calculate the prediction position by using the past three measurements. Therefore, the filter cannot respond quickly for the target that changes direction instantaneously. Figure 17 illustrates that the 3PCL IMM filter has less estimation error than the linear filter when the target turns to the change direction. Compared with the 3PC filter, the 3PCL IMM filter has a relatively small oscillation in the estimated response. Figure 18 shows that the 2PCT IMM filter proposed in this paper has a smaller estimation error than the 3PCL IMM filter when the target is to change the direction of motion. In addition, the oscillation of the estimated response is also very slight. Figure 19 shows the comparison of the estimation errors under the condition of Scenario 2 for different type filters. It shows that the linear filter has a larger estimation error than other estimators. After the target suddenly changed the direction at each way point, the 3PC circular filter has a larger estimation error than 3PCL and 2PCT IMM filters, and the estimation error variations are more drastic. In addition, we observed that the 3PCL IMM filter has a larger estimation error than the 2PCT IMM filter, and the variation of the estimation error is also large, which means that the 2PCT IMM filter has a better estimation performance than the 3PCL IMM filter.

Table 1 shows the estimation performances of maximum estimation error (ME) and RMSE. It can be seen that the estimation RMSE value of the linear filter is larger than that of the other three types of filters, regardless of Scenario 1 or Scenario 2; and the estimation RMSE value of the 3PCL IMM filter is smaller than the 3PC filter. The 2PCT IMM filter has the smallest estimation RMSE value and the smallest ME. This confirms that the proposed 2PCT IMM filter has better estimation performance. In Scenario 2, we observed that the 3PC filter has the largest ME among the four types of filter. This phenomenon is caused by excessive oscillation of estimation response. It also demonstrates that using a single circular filter can cause significant estimation errors under certain maneuvering conditions, which may make it difficult to effectively track the target.

## 6. Conclusions and Future Works

Small-sized rotary phased array radars are widely used due to their light weight, low price, and high mobility. However, they must use the TWS method to track the target under low update rate conditions. Therefore, maintaining a good tracking performance for maneuvering targets under low update rate conditions has become an important issue. This study proposed a circular prediction algorithm (3PC) based on the use of three previously known positions to predict the next-step unknown position. In addition, it verifies the ability of a hybrid filter (3PCL) designed by integrating a linear estimator with a circular estimator to effectively track maneuvering targets. It noted, however, that the method may result in slower estimated responses, due to excessive prior information requirements. In this paper, we derived an algorithm that can estimate the future unknown position based on two previously known positions. The proposed algorithm consists of two parts, a 2 points circular predictor (2PC) and a 2 points tangent predictor (2PT); and a new type of IMM filter combining these two algorithms was designed. The simulation results show that under low update rate conditions, the 2PCT IMM filter proposed in this paper has a faster and more stable estimation response than the 3PCL IMM filter.

The algorithm proposed in this work mainly considers how to obtain a filter value that is closer to the real target position under a low update rate (*t*_s_ > 2 s). The predicted value is not easy to obtain for a low update rate radar system, and the predicted error is usually much larger than the measurement error. Therefore, this paper does not consider the influence of the measurement error on the filtering performance of the system and instead focuses on developing an algorithm that can obtain a better predicted value under a low update rate. The effects of measurement noise, update rate and threshold values on filtering performance can be studied in future research for fast update rate radar systems.

## Figures and Tables

**Table 1:** Estimation performance of different filters.

Scenario	Linear Filter		3 Points Circular Filter (3PC)		3PCL IMM Filter		2PCT IMM Filter	
	ME (m)	RMSE (m)	ME (m)	RMSE (m)	ME (m)	RMSE (m)	ME (m)	RMSE (m)
1	715.15	461.09	684.05	287.58	422.82	214.45	289.55	152.04
2	2086.18	955.23	2126.09	854.36	1358.24	620.75	1012.30	438.24
